# Congenital Duodenal Obstruction in Neonates: Over 13 Years' Experience from a Single Centre

**DOI:** 10.21699/jns.v5i4.461

**Published:** 2016-10-10

**Authors:** Parveen Kumar, Chiranjiv Kumar, Prince Raj Pandey, Yogesh Kumar Sarin

**Affiliations:** Department of Pediatric Surgery, Maulana Azad Medical College, New Delhi, India.

**Keywords:** Duodenum, Obstruction, Atresia, Malrotation

## Abstract

Aim: To study the prevalence of associated anomalies with neonatal duodenal obstruction and factors impacting short-term survival.

Material and methods: Records of 31 neonates with neonatal duodenal obstruction could be retrieved and analyzed for a 13.5-year-period (October 2003-May 2016). M:F ratio was 1.58:1. The mean birth weight was 2.15 kg; 12 patients were preterm. Etiologies included duodenal atresia (n=23), duodenal web (n=8) and malrotation of gut (n= 6).

Results: Associated anomalies were seen in 19/31: Down's syndrome (n=6), anorectal malformation (ARM) (n=5), annular pancreas (n=5), cardiac anomalies (n=4), esophageal atresia with trachea-esophageal fistula (EA with TEF) (n=3). Mortality in the series was 22.5%; 5 deaths and 2 patients left against medical advice in moribund state (hidden mortality). Mortality in associated anomalies group was 5/19; and 2/12 in the no anomalies group, though this difference was not statistically significant (p=0.676). Similarly, low birth weight (LBW) did not have impact on survival (p=0.639) but preterm status had highly significant p value (<0.001).

Conclusion: Duodenal atresia was the commonest cause of neonatal duodenal obstruction. Associated anomalies were noted in 61% patients, Down's syndrome being the most frequent. These anomalies did not have any significant impact on the survival, nor did LBW. Preterm status had significant impact on prognosis.

## INTRODUCTION

Congenital duodenal obstruction (CDO) is amongst the commonest anomalies in newborns. [1] It affects about one in 2500 to 10000 live births, and nearly half of all cases of neonatal intestinal obstruction. [2,3] It may be either complete or partial and causes are classified as intrinsic (duodenal atresia and web) or extrinsic (malrotation with Ladd's band and annular pancreas). [4-7] Survival of neonates has improved commendably because of advances in surgical management, intensive care medicine, and postoperative nutritional support. We reviewed our experience in managing CDO in 31 neonates over 13.5 years.

## MATERIALS AND METHODS

A retrospective study was performed by retrieving the medical records of neonates who underwent surgery for congenital duodenal obstruction in the Department of Pediatric Surgery in a public tertiary-care hospital. This group was studied in detail with respect to the demographic details, antenatal diagnosis, clinical presentation, associated anomalies, surgical procedure, postoperative complications and outcome including mortality of these patients. 

## RESULTS

Demographic Details: A total of 31 neonates with congenital duodenal obstruction including 23 patients of duodenal atresia and 8 patients with duodenal web, were managed over a period of 13.5 years (October 2003 to May 2016). The age distribution of neonate included from day 1 to day 28 of life. Most frequent age of presentation was day 1 of life (12 neonates). Twelve patients (38.7%) were premature (31-36 weeks). There was male sex predilection (out of 31 neonates, 19 were males and 12 were females) with the M: F ratio being 1.58:1.

Antenatal Diagnosis: Though majority of the pregnancies were supervised, an antenatal diagnosis of duodenal obstruction on ultrasonography (Fig. 1) was made in only two patients. One mother was diagnosed to have polyhydramnios.

**Figure:**
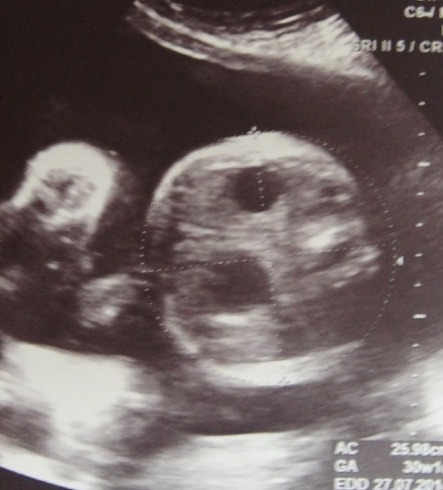
Figure 1: USG- showing fetal double bubble sign with polyhydramnios.

Clinical Presentation: The commonest presentation was vomiting after breast feeds. Five patients presented with ARM. Three patients with associated EA with TEF presented with frothing from the mouth. The mean birth weight was 2.15 kg. Twenty two neonates were low birth weight (LBW)(70.9%). 

Associated Anomalies: The frequencies of associated anomalies are shown in Table 1. 

**Figure:**
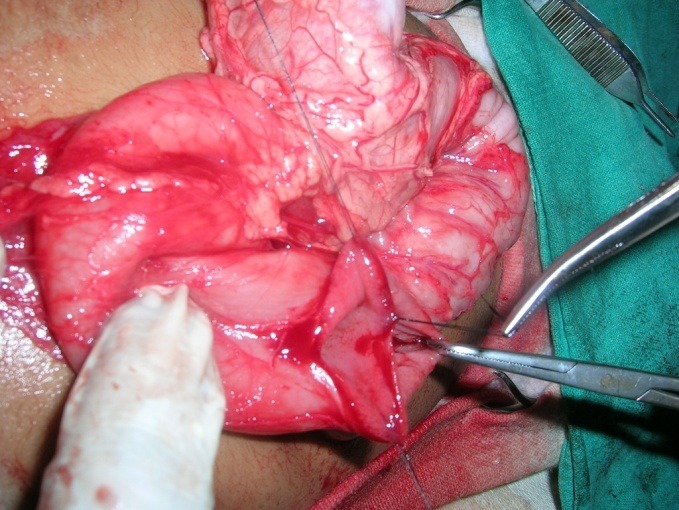
Figure 2: Lateral duodenotomy with excision of the duodenal web.

Radiological Investigation: In all of the cases, plain abdominal roentgenograms were diagnostic (double-bubble appearance). 

Surgery: Emergency operation was done in 17, while 14 were operated electively. After an initial assessment of associated congenital anomalies and hemodynamic stabilization, the patients underwent laparotomy through a right upper quadrant transverse incision. The etiology for congenital duodenal obstruction was assessed in intra operative period. Etiologies included duodenal atresia (n=23), duodenal web (n=8) and malrotation of gut (n= 6). All patients of duodenal atresia, duodenal webs and annular pancreas underwent exploratory laparotomy with trans-anastomotic tube (TAT) placement at end of procedure. 

In patient with duodenal atresia and annular pancreas, Kimura's diamond shaped duodeno-duodenostomy was performed. A lateral duodenotomy with excision of the obstructive membrane was done in duodenal web patients (Fig. 2). The duodenotomy was closed transversely using 6/0 or 5/0 interrupted delayed absorbable sutures in a single layer. The location of the web was between the first and second parts of duodenum in all except two, in whom the membrane was located at the duodeno-jejunal flexure. Ladd's procedure was done for associated malrotation in six patients. 

**Figure:**
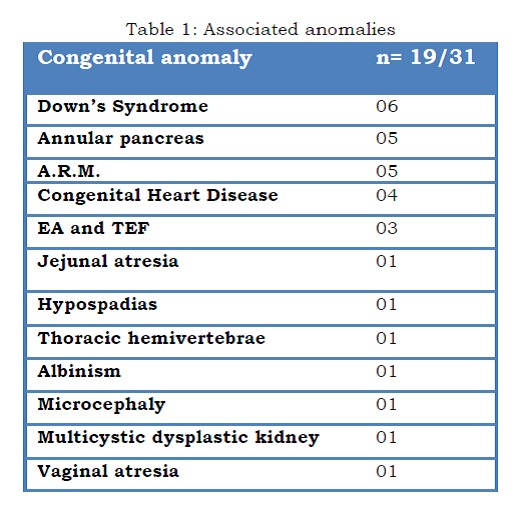
Table 1: Associated anomalies

No significant intra-operative surgical or anesthetic difficulties were encountered. It was possible to visualize the exact location of the membrane in the duodenum by careful inspection during surgery. Post operative ventilator support and supportive intensive care were required in seven patients. TAT tube feeds were started on postoperative day (POD) 5 in 10 (32.25%), on POD-6 in 7 (22.58%), on POD-4 in 6 (19.35%) and rest later. 

In postoperative period, 12 (38.7%) had sepsis and 1 (3.2%) each had intraventricular hemorrhage, congestive heart failure, short bowel syndrome and meningitis. One patient had accidental removal of TAT tube on POD 3 but managed conservatively. Two needed re-operations, 1 for associated large sliding hiatal hernia and 1 for mid ileal perforation. 

Five patients (16.12%) were expired in postoperative period and 2 (6.45%) left against medical advice in moribund state (hidden mortality). Total mortality in series was 22.5 %. Mortality in associated 'anomalies group' was 5/19, and 2/12 in the 'no anomalies' group, though this difference was not statistically significant (p=0.676) .Similarly, low birth weight (LBW) did not have impact on survival (p=0.639) but preterm status had highly significant p value (<0.001).

## DISCUSSION

Congenital duodenal obstruction can present at variable age. Complete obstructive lesions like duodenal atresia present earlier as compared to partial obstruction like duodenal web, malrotation and annular pancreas. [3] Most frequent age of presentation for duodenal atresia was day 1 and day 2 for duodenal web. 

Duodenal atresias (DA) were observed in 23(74.1%) patients and were the most common cause of obstruction. Out of 12 preterm babies, 11 were associated with duodenal atresia and 1 had duodenal web. It could be explained by increase chances of preterm delivery in view of polyhydramnios associated with duodenal atresia. Escobar had similar findings in their study with 169 neonates, where 37% were preterm [5] and Rattan et al study shows out of 38 neonates 10 (26.3%) were preterm.[6]

In more than half of the patients with duodenal atresia, associated anomalies and syndromes are present. Down syndrome is present in 30% of cases, malrotation in 20%, and congenital heart diseases in 20% of cases; nevertheless, other congenital anomalies of alimentary tract are also present in these patients[9]. In 2/3rd patients, the associated anomalies occur in isolation, whereas multiple anomalies occur in 1/3rd of patients. Although presence of Down syndrome in patients of duodenal atresia does not affect survival, the presence of multiple anomalies may alter the final outcome.[5, 9]

In the current era, majority of cases are diagnosed in antenatal period but in our series only 2 patients had antenatal diagnosis as most of the obstetrics and anomaly scan are still being done by the treating obstetrician thus missing the findings of CDO. 

Surgical approach is dictated by type of the anomaly. Diamond shaped anastomosis as popularized by Kimura was attempted in all duodenal atresia cases.[10] In patients with duodenal web, lateral duodenotomy was done and web was excised with electrocautery leaving the medial part, avoiding injury to the ampulla of Vater. A proximal 'mega-duodenum' (duodenal diameter of 5 cm or more) may require imbrications or a tapering duodenoplasty procedure to avoid prolonged duodenal ileus. [11] Problem faced in initiating the oral feeds due to the gross duodenal dilatation leading to late discharge of pts. In our series though average discharge was at 13 to 14 days but the exact details regarding the oral feed toleration was not mentioned.

Escobar et al found 6% of late mortality attributed to associated anomalies, central nervous system bleeding, pneumonia, and anastomotic disruption etc. [5] Rattan et al had reported mortality of 11 (13.5%) out of 81 neonates[6], which is similar to the mortality rate in our group. Zamir et al had reported mortality of 58% in a series from Pakistan. [7] In a review of 287 neonates, Chen et al observed overall mortality of 5.9% .[8] In western counties where antenatal supervision is good and neonates are diagnosed early, postoperative outcome of duodenal obstruction is much better than developing countries.[12,13]

## Conclusion

Duodenal atresia was the commonest cause of neonatal duodenal obstruction. Associated anomalies were noted in 61% patients, Down's syndrome being the most frequent. These anomalies did not have any significant impact on the survival, nor did LBW but preterm status did. With increased ICU care and meticulous surgery, most of these patients have good survival. 

## Footnotes

**Source of Support:** None

**Conflict of Interest:** None
